# Letter from Prof. Keyser, Dean of the Medico-Chirurgical College, Philadelphia

**Published:** 1886-10

**Authors:** Peter D. Keyser

**Affiliations:** Dean; Medico-Chirurgical College of Philadelphia


### CORRESPONDENCE.

MEDICO-CHIRURGICAL COLLEGE OF PHILADELPHIA, September 25, 1886.

EDITOR DANIEL'S TEXAS MEDICAL JOURNAL:

DEAR SIR—It has been the purpose of the Faculty of the Medico-Chirurgical College to carry on its work in harmony with all, for the higher education of medical students, without discussion with any School. But, when remarks are made which are neither fair, just nor truthful, it makes it necessary that a reply should be given.

Professor Bartholow, Dean of the Jefferson Medical College, in his letter to you, as published in your JOURNAL for September, makes the charge that "they" (the Medico-Chirurgical College) are abusing the Jefferson Medical College. In the question he asks, "Why do they find it necessary to abuse that [the Jefferson Medical College] institution in their attempt to found a new one?"

The Faculty of the Medico-Chirurgical College desire me to state that nothing has ever been said or done derogatory to the Jefferson Medical College. If Professor Bartholow has heard that any such action has been taken, his information is from an improper and incorrect source.

An attempt to found a new School is not being made, but to bring to the front one that has been working for the principle of higher, longer, and more thorough education, for some years. In doing this, it has been with the warmest and most friendly feelings for all of the other schools, and with the belief that those Faculties would see that the establishment of other schools in this city must inure to their benefit also; for the more schools, the greater the attraction for students, justifying the old reputation of Philadelphia as the great medical center of the country.

The Faculty desire, also, to say that they are not attempting to build up a School on a sort of "fashionable programme." They are simply making an effort to comply with the expressed general demands of the profession throughout the country. If, by complying with these demands for higher education, our curriculum is to be dubbed a "fashionable programme," we are glad, and feel proud to be in the *fashion*. Yours very truly,

PETER D. KEYSER, Dean.

